# Supplementary material for: Determining the Development Strategy and Suited Adoption Paths for the Core Competence of Shared Decision-Making Tasks through the SAA-NRM Approach
Source: Int J Environ Res Public Health. 2022 Oct 15;19(20):13310. doi: 10.3390/ijerph192013310 (PMC9602580; doi:10.3390/ijerph192013310)
Supplement: Supplementary file 1 [file ijerph-19-13310-s001.zip › ijerph-1927994-supplementary.pdf]

Supplementary Materials

1. The SAA Analysis

The first adoption step is to improve those aspects (i.e., skill and practice (SP) and concept and evaluation (CE)) falling into the third quadrant (L, L), indicating the low satisfaction index and low attention index. The second adoption step is to enhance those aspects (i.e., communication and relationship (CR), joint information and decision making (JM)) that fall into the first quadrant (H, H), showing the high satisfaction index and high attention index, as shown in Table S1 and Figure S1.

Table S1. The SAA analysis of the competence assessment.

Aspects	SI		AI		(SI, AI)
	MS	SS	MA	SA	
Concept and evaluation (CE)	7.672	−0.518	7.951	−1.039	(L, L)
Skill and practice (SP)	7.542	−1.137	7.987	−0.673	(L, L)
Communication and relationship (CR)	7.913	0.640	8.142	0.899	(H, H)
Joint information and decision making (JM)	7.992	1.015	8.133	0.813	(H, H)
Average	7.780	0.000	8.053	0.000	
Standard deviation	0.209	1.000	0.098	1.000	
Maximum	7.992	1.015	8.142	0.899	
Minimum	7.542	−1.137	7.951	−1.039	

Note 1: (H, H) means the aspect/criteria of high satisfaction and high attention, (L, H) means the aspects / criteria of low satisfaction and high attention. (L, L) means aspects/criteria of low satisfaction and low attention, and (H, L) means the aspects/criteria of high satisfaction but low attention. Note 2: MS, SS, MA, and SA means satisfaction (MS), standardized satisfaction (SS), mean attention (MA), and standardized attention (SA), respectively. Notes 3: The development strategies include four types: development strategy A (situation maintaining), development strategy B (direct strengthening), development strategy C (sequentially strengthening), and development strategy D (circumstance watching).

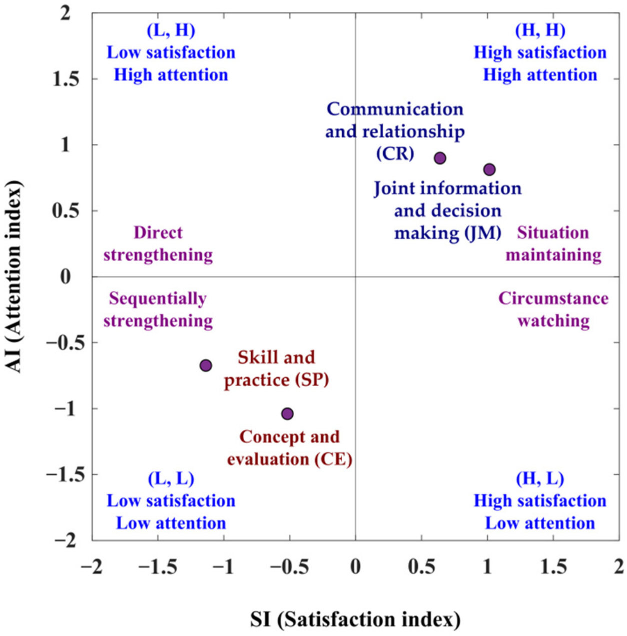

Figure S1. The position map based on SAA (satisfaction and attention analysis) approach.

2. *DEMATEL Approach*

The DEMATEL technique is divided into five stages in this study: (1) estimate the original average matrix, (2) compute the direct influence matrix, (3) determine the indirect influence matrix, (4) count the full influence matrix, and (5) establish the NRM graph.

(1) Estimate the original average matrix

The original average matrix is as shown in Table S2. The influence of "skill and practice (SP)" on "communication and relationship (CR)" is 2.847, i.e., "medium influence." The influence of "concept and evaluation (CE)" on "joint information and decision making (JM)" is 2.576, which also indicates "medium influence," as shown in Table S2.

Table S2. The original average influence matrix (A).

Aspects	CE	SP	CR	JM	Total
Concept and evaluation (CE)	0.000	2.712	2.720	2.576	8.008
Skill and practice (SP)	2.661	0.000	2.847	2.805	8.314
Communication and relationship (CR)	2.610	2.771	0.000	2.737	8.119
Joint information and decision making (JM)	2.568	2.678	2.797	0.000	8.042
Total	7.839	8.161	8.364	8.119	-

(2) Compute the direct influence matrix

From Equations (1) and (2), the D (direct influence matrix) can be attained by A (initial average influence matrix), as illustrated in Table S3. As shown in Table S4, the sum of rows and columns for "communication and relationship (CR)" is 1.971, the most critical influence aspect. On the contrary, the sum of rows and columns for "concept and evaluation (CE)" is 1.895, which is the least influential aspect.

Table S3. The direct influence matrix (D).

Aspects	CE	SP	CR	JM	Total
Concept and evaluation (CE)	0.000	0.324	0.325	0.308	0.957
Skill and practice (SP)	0.318	0.000	0.340	0.335	0.994
Communication and relationship (CR)	0.312	0.331	0.000	0.327	0.971
Joint information and decision making (JM)	0.307	0.320	0.334	0.000	0.961
Total	0.937	0.976	1.000	0.971	-

Table S4. The degree of direct influence.

Aspects	Sum of row	Sum of column	Sum of row and column	Importance of influence
Concept and evaluation (CE)	0.957	0.937	1.895	4
Skill and practice (SP)	0.994	0.976	1.970	2
Communication and relationship (CR)	0.971	1.000	1.971	1
Joint information and decision making (JM)	0.961	0.971	1.932	3

(3) Determine the indirect influence matrix

The indirect influence matrix can be obtained from Equation (3) and presented in Table S5.

Table S5. The indirect influence matrix (ID).

Aspects	CE	SP	CR	JM	Total
Concept and evaluation (CE)	7.710	7.867	8.014	7.845	31.436
Skill and practice (SP)	7.850	8.171	8.236	8.057	32.314
Communication and relationship (CR)	7.715	7.946	8.177	7.919	31.757
Joint information and decision making (JM)	7.662	7.894	8.035	7.943	31.534
Total	30.937	31.878	32.462	31.764	-

(4). Count the full influence matrix

T (full influence matrix) and the degree of full influence can be attained by Equation (4) or (5) and presented in Table S6 and Table S7, respectively. As indicated in Table S8, the communication and relationship (CR) aspect was the highest degree of total influence ($d3 + r3 = 66.188$). The concept and evaluation (CE) aspect is the highest net influence ($d1 - r1 = 0.520$). The other net influences are in the following order: the aspects of skill and practice (SP) ($d2 - r2 = 0.454$), joint information and decision making (JM) ($d4 - r4 = -0.240$), and communication and relationship (CR) ($d3 - r3 = -0.734$).

Table S6. The full influence matrix (T).

Aspects	CE	SP	CR	JM	*d*
Concept and evaluation (CE)	7.710	8.191	8.339	8.153	32.393
Skill and practice (SP)	8.168	8.171	8.576	8.392	33.307
Communication and relationship (CR)	8.027	8.277	8.177	8.246	32.727
Joint information and decision making (JM)	7.969	8.214	8.369	7.943	32.495
r	31.874	32.853	33.461	32.734	-

Table S7. The degree of full influence.

Aspects	*d*	*r*	*d* + *r*	*d* – *r*
Concept and evaluation (CE)	32.393	31.874	64.267	0.520
Skill and practice (SP)	33.307	32.853	66.159	0.454
Communication and relationship (CR)	32.727	33.461	66.188	−0.734
Joint information and decision making (JM)	32.495	32.734	65.229	−0.240

(5). Establish the NRM graph

Equation (9) yields the net influence matrix in Table S8. We are using the values of ($d + r$) and ($d - r$) from Table S7 as the X and Y values to draw the NRM graph, respectively, as illustrated in Figure S2. The concept and evaluation (CE) aspect is the primary aspect with net influence, while the communication and relationship (CR) aspect is the primary aspect being influenced as shown in Figure S2 and Table S8.

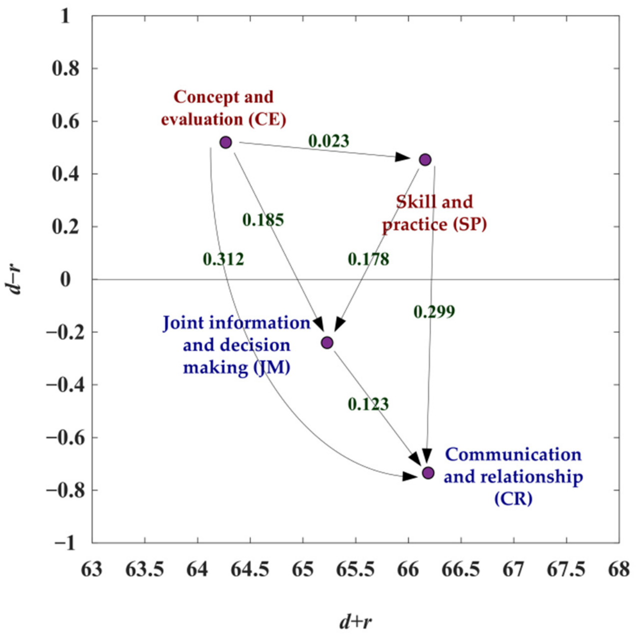

Figure S2. The NRM for competency development of SDM tasks.

Table S8. The net influence matrix for competence development of SDM tasks.

Aspects	CE	SP	CR	JM
Concept and evaluation (CE)	-			
Skill and practice (SP)	−0.023	-		
Communication and relationship (CR)	−0.312	−0.299	-	
Joint information and decision making (JM)	−0.185	−0.178	0.123	-
